# CFTR and Wnt/beta-catenin signaling in lung development

**DOI:** 10.1186/1471-213X-8-70

**Published:** 2008-07-06

**Authors:** J Craig Cohen, Janet E Larson, Erin Killeen, Damon Love, Ken-Ichi Takemaru

**Affiliations:** 1 The Brady Laboratory, Section of Neonatology, Department of Pediatrics, Stony Brook University, School of Medicine, Stony Brook, New York, USA; 2 Department of Pharmacological Sciences, Stony Brook University, School of Medicine, Stony Brook, New York, USA

## Abstract

**Background:**

Cystic fibrosis transmembrane conductance regulator (CFTR) was shown previously to modify stretch induced differentiation in the lung. The mechanism for CFTR modulation of lung development was examined by *in utero *gene transfer of either a sense or antisense construct to alter CFTR expression levels.

The BAT-gal transgenic reporter mouse line, expressing β-galactosidase under a canonical Wnt/β-catenin-responsive promoter, was used to assess the relative roles of CFTR, Wnt, and parathyroid hormone-related peptide (PTHrP) in lung organogenesis. Adenoviruses containing full-length CFTR, a short anti-sense CFTR gene fragment, or a reporter gene as control were used in an intra-amniotic gene therapy procedure to transiently modify CFTR expression in the fetal lung.

**Results:**

A direct correlation between CFTR expression levels and PTHrP levels was found. An inverse correlation between CFTR and Wnt signaling activities was demonstrated.

**Conclusion:**

These data are consistent with CFTR participating in the mechanicosensory process essential to regulate Wnt/β-Catenin signaling required for lung organogenesis.

## Background

Organogenesis in the lung requires the sequential development of the large airways, bronchioles and alveoli. The lung begins as a simple structure with increasing complexity that is necessary for efficient air exchange at maturity. Recently, several laboratories have demonstrated that this complex process involves the promotion of mechanical stretch [1-4]. Specifically, muscle contractions of the large airways compress amniotic fluid in the fetal lung generating a pressure gradient that is converted to biochemical signals necessary for cell differentiation.

Mechanical force modulation of biochemical processes is a well known phenomenon that is mediated by multimeric proteins [4,5]. Mechanicosensing and the genes involved, however, are less well known. Sensing molecules such as integrins, ion channels, and kinase-linked receptors have been implicated in changes in gene expression related to stretch [6]. Most of the evidence from these studies implicates vascular responses to stretch in hemodynamic stasis, although little is known about these sensors in fetal organogenesis.

Recently, control of muscle contractions via the Rho kinase-dependent and independent pathways was shown to be important in lung development [3]. In particular, the cystic fibrosis transmembrane conductance regulator (CFTR) was found to influence muscle contractions in lung development via a Rho kinase-independent mechanism involving ATP and calcium [7]. In other work, we and others demonstrated that the canonical Wnt/β-catenin pathway plays crucial roles in lung organogenesis [8,9]. These studies utilized our laboratory's transient *in utero *gene transfer technique [10] with sense and antisense constructs to modulate gene expression [11-16]. This technique has the advantage in lung development studies by transiently targeting the developing respiratory epithelium for temporal modulation of gene expression without any inflammatory consequences and no premature expression leakage.

In the study reported here the role of CFTR in downstream regulation of lung differentiation effectors was examined. Specifically, the relationship between CFTR function and Wnt/β-catenin signaling in lung development was examined. Previous studies have suggested that Wnt signaling via a stretch-induced mechanism involving parathyroid hormone-related peptide (PTHrP) is required for normal lung development [17-20]. Therefore, we used our experimental system to determine if altering CFTR expression has downstream effects on Wnt and PTHrP which would provide further evidence of CFTR involvement in mechanicosensing necessary for normal lung development.

## Results

### CFTR influences Wnt/β-catenin signaling

BAT-gal reporter mice express β-galactosidase under the control of Wnt//β-catenin-responsive elements [24]. To evaluate the role of CFTR in modulating the Wnt signaling cascade, fetuses from time pregnant BAT-gal mice were injected at E15, E16, and E17 via the amniotic fluid using our established techniques [11,23,25]. Recombinant adenoviruses with either eGFP (AdCMVeGFP; control), CFTR (Av1CF2; [12,13,15]), or antisense CFTR (AdCMVascftr; [22] were used to alter levels of CFTR gene expression in the developing lung. Tissues were harvested at 24 hours post-gene transfer, homogenized (3–4 fetuses/pool), and β-galactosidase enzyme and total protein assays performed and the enzymatic activity was expressed as units/μg protein.

**Figure 1:**
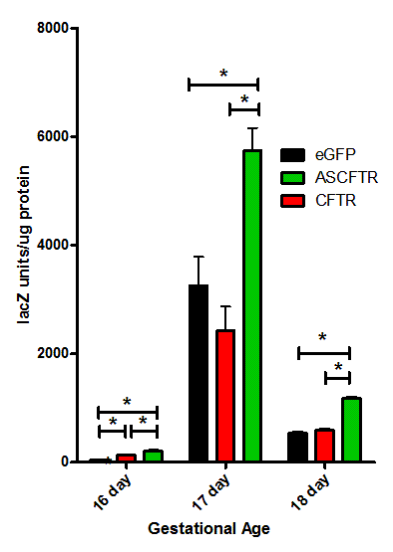
**β-galactosidase activity following *in utero *modification of CFTR expression**. Bat-gal mice were injected at E15, E16, or E17 with adenoviruses expressing eGFP (control), antisense CFTR (ASCFTR), or CFTR and lungs harvested 24 hours later. Tissue was homogenized and β-galactosidase enzyme assays and total protein determinations performed. * p < 0.05.

BAT-gal expression during normal lung development was found to be highly regulated. At E16 little if any Wnt/β-catenin-dependent β-galactosidase activity was detected in control tissues (Fig. 1). Enzyme activity peaked at E17 and then decreased by E18. CFTR over expression resulted in a small but significant (p < 0.05) increase in BAT-gal expression at E16. At E17 there was a non-significant decrease in activity in the CFTR-treated animals, and by E18 there was no difference between control and CFTR-treated animals. In contrast, antisense knockdown of CFTR showed highly significant (p < 0.01) increases in BAT-gal activity at E16, E17, and E18 when compared to either control or CFTR-treated animals consistent with a relationship between CFTR expression and canonical Wnt signaling between E16–E18 in the mouse lung.

**Figure 2:**
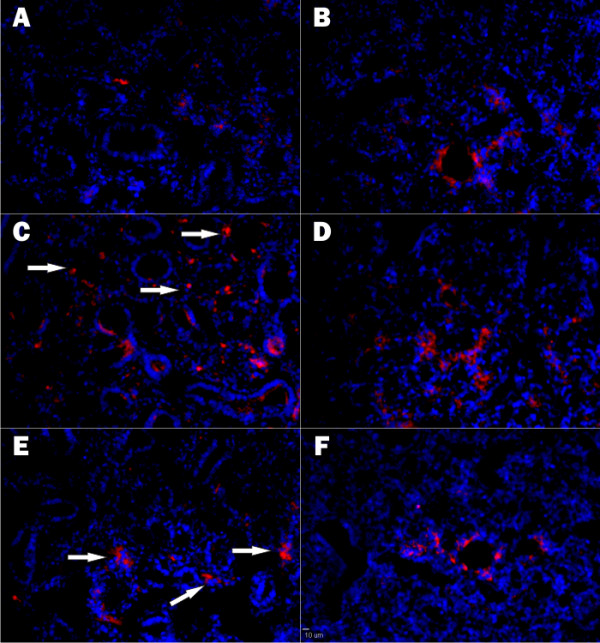
**Distribution of β-galactosidase expression in embryonic lungs**. Immunohistochemistry was used to identify cells expressing β-galactosidase in Bat-gal mouse fetal lungs from either E16 (Panels A, C, & E) or E17 (Panels B, D, & F). 24 hours prior to harvest animals were treated with either AdCMVeGFP (control; panels A & B); AdCMVascftr (antisense CFTR; Panels C & D); Av1CF2 (CFTR; Panels E & F). 10 μm scale bar is presented in Panel F.

The distribution by fluorescent immunohistochemistry of β-galactosidase expression was significantly different in fetuses treated at E15 and examined at E16 (Fig. 2A, C, D), AdCMVeGFP, control, lungs showed little or no expression as would be expected from the enzyme assay data presented in Fig. 1. Antisense CFTR treated lungs, which had the largest increase in β-galactosidase enzyme activity, exhibited expression in randomly distributed individual cells (arrows; Fig. 2C). In contrast, CFTR over expression, which showed a smaller but significant increase in enzyme activity, exhibited increased clusters of β-galactosidase expressing cells (arrows; Fig. 2E). In fetuses treated at E16 and examined at E17, normal, control, and CFTR over expression (Fig. 2B and 2F), β-galactosidase was seen around small airways with some expression in the parenchyma consistent with CFTR accelerating the normal developmental Wnt/β-catenin signaling. In contrast, inhibition of CFTR expression at E16 resulted in further expansion of β-galactosidase positive cell populations to the parenchyma (Fig. 2D). Thus, consistent with the results of Fig. 1, Wnt/β-catenin signaling patterns are dependent upon CFTR expression levels. Over expression facilitated the normal pattern for Wnt/β-catenin and antisense inhibition of CFTR resulted in an abnormal increase and distribution in foci of β-galactosidase positive cells.

### Developmental programming of PTHrP expression by CFTR

Based on the previously demonstrated relationship between mechanical stretch, Wnt signaling and PTHrP expression [17,19,20,26] and our previous work on CFTR that has shown that it modulates stretch to influence gene regulation in the developing lung [7], we investigated the relationship between CFTR-dependent stretch and PTHrP levels.

**Figure 3:**
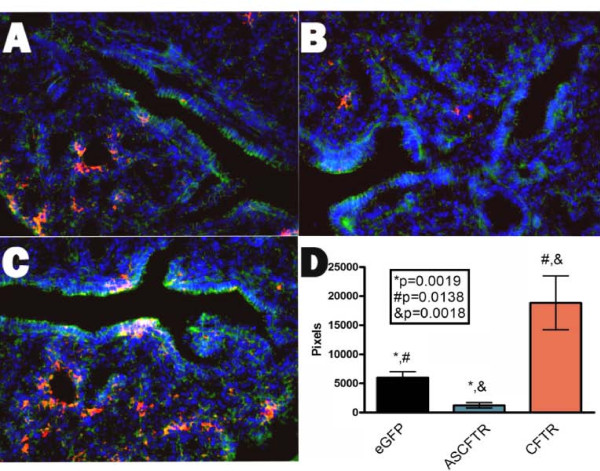
**PTHrP in lung development**. Bat-gal mice were injected with either AdCMVeGFP (Panel A), AdCMVascftr (Panel B), or Av1CF2 (Panel C; Adenovirus CFTR) at E15 and tissues harvest at E16. Tissues were stained with DAPI (Blue), Phalloidin (Green), and immunofluorescent analysis of PTHrP (Red). Slidebook software was used for pixel counting of PTHrP in a series of slides (Panel D).

Immunohistochemistry was used to visualize PTHrP expression at E17, the time when CFTR expression had the greatest effect on BAT-gal reporter expression (Fig. 1). In control animals low levels of PTHrP were detected in the developing parenchyma (Fig. 3, Panel A). In marked contrast, down-regulation of CFTR with the antisense construct resulted in a dramatic decrease in PTHrP immunostaining (Panel B), whereas CFTR over expression (Panel C) resulted in a significant increase in PTHrP expression in comparison to both control- and antisense CFTR-treated lungs (Panel C). Quantitative evaluation of PTHrP showed statistically significant differences between control-, antisense CFTR-, and CFTR-treated lungs (Panel D). Thus, there was a direct correlation between CFTR expression and PTHrP levels in the developing lung at E17.

## Discussion

CFTR is expressed at high levels during specific periods of lung organogenesis and expression subsequently decreases by 75 fold as the lung matures [27,28]. This pattern of expression suggests a role for this gene in normal lung development. The finding that CFTR has a role in mechanical stretch which is necessary for lung organogenesis [7] provides a possible mechanism for this gene in lung development.

Specifically, our findings of a direct correlation between CFTR expression and PTHrP levels (Fig. 3) in developing lung and the inverse correlation with Wnt/β-catenin signaling (Fig. 1 &2) are consistent with the mechanical stretch-dependent development of surfactant production[29]. These data collectively reveal a possible mechanism for how a chloride channel, CFTR, could have a global effect on lung development. By directly effecting mechanical stretch necessary for generating lung complexity this chloride channel could have an essential role in normal lung organogenesis.

**Figure 4:**
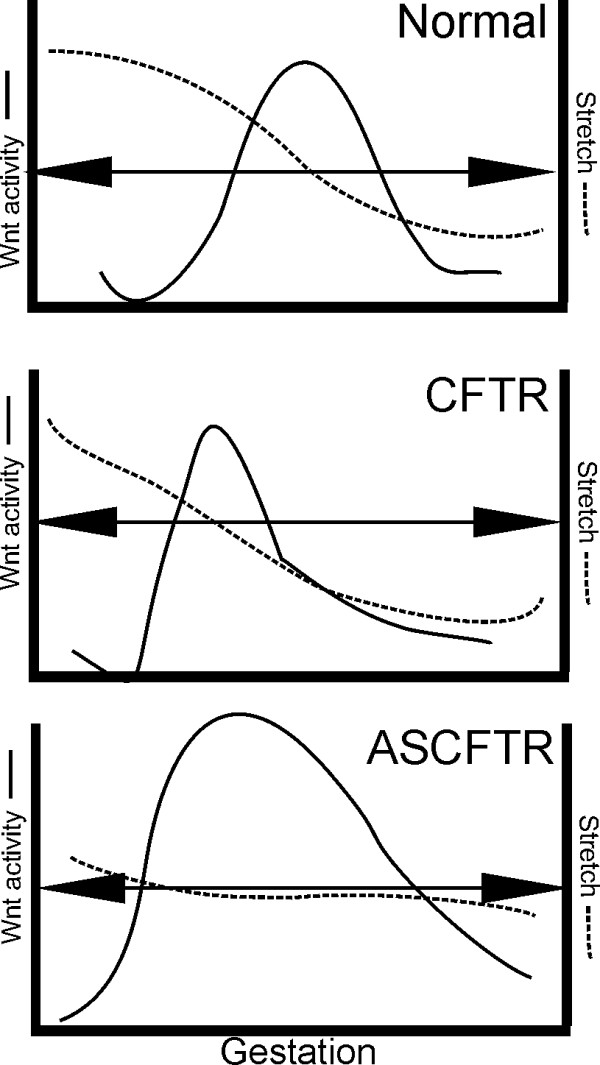
**Relationship between stretch and Wnt**. Changes in CFTR levels such that the nexus (Arrow) for maximal stretch-dependent Wnt activation is reached sooner and transit time across this stimulus altered.

In the normal Bat-gal mouse lung Wnt expression peaked at a specific time (Fig. 1) in lung development that may be defined by the total induced stretch (Fig. 4, Arrow). If CFTR increases stretch as shown previously [7] and illustrated in Fig. 4, then one would predict that the slope of the change in stretch would be increased. Thus, Wnt expression would peak earlier for a shorter time and lower level. Antisense CFTR, however, would lower the slope of the stretch effect, prolonging Wnt activation and increasing its activity. Thus, the changes observed in Fig. 1 can be explained by a change in CFTR-dependent, stretch-induced regulation of Wnt. This interpretation is consistent with the previous work by the Torday and Rehan group showing the relationship between stretch and Wnt [19,26]

Our previous studies examining the effects of *in utero *CFTR gene transfer on lung growth and development showed that over expression of CFTR resulted in increased bronchial cell differentiation and proliferation, decreased alveolar complexity, and increased stretch [7,12]. In this paper we demonstrated that CFTR over expression increases PTHrP (Fig 3); and decreased CFTR expression dramatically increased Wnt signaling (Fig 1, 2). If one examines all these CFTR-dependent effects both previously published [7,12,13,15,22,30] and current data provided here, CFTR, a chloride channel, has many, seemingly independent, affects on lung organogenesis. However, stretch was previously shown by Torday and Rehan [17,19,26,29,31] to correlate directly with PTHrP which controls a cascade in which lower PTHrP (from lower stretch) increases Wnt activation that leads to increased bronchial cell differentiation and proliferation and decreased alveolar complexity. Thus, it is possible to correlate all the effects seen independently by altering CFTR levels in the fetal lung with a direct effect of CFTR on stretch that then modifies the Torday-Rehan PTHrP-Wnt pathway for terminal differentiation of the lung. Initially, mechanical stretch promotes proximal airway development but with growth, distal airway differentiation results in decreased pressure and Wnt-dependent alveolar differentiation and complexity. Our results correlated with those of Torday and Rehan [17-20,31-34] suggest that CFTR could play a central role in this process along with other genes that affect mechanical stretch including Rho kinase [35] and ENaC [36,37].

Delineation of the exact role of CFTR in this process requires further investigation. However, several recent findings may suggest a mechanism. Treharne et al. [38] recently showed that CFTR function was linked to casein kinase 2, a rather promiscuous protein kinase. In addition, CFTR function is known to be associated with actin filaments [39,40] and recent work has shown that a cAMP/protein kinase A-dependent annexin 2 S100A10 complex with CFTR also affects its function [41]. These observations provide increasing evidence that the CFTR gene product plays important roles beyond that of a chloride channel and that it is part of a complex of molecules within the cytoskeleton of the cell that have a high potential for kinase-mediated gene regulation. Therefore, we speculate that stretch of the cytoskeleton could be the signal to modulate CFTR function and mechanical stretch. Thus, CFTR could function as a mechanicosensor essential for lung organogenesis.

## Conclusion

During late lung development CFTR probably acting through a mechanicosensory pathway regulates Wnt/β-Catenin signaling.

## Methods

### BAT-gal Wnt/β-Catenin Reporter Mice

Generation and characterization of the BAT-gal mice have been previously described [21]. E1 was defined as the day that the vaginal plug was observed.

### In Utero Gene Transfer

*In utero *gene transfer used three first generation adenovirus vectors. Av1CF2 is a recombinant with the CFTR gene (Genetic Therapy, Inc), AdCMVascftr is an antisense CFTR construct previously described [22] and AdCMVeGFP with the green fluorescent protein gene (J. Kolls, Univ. Pittsburgh) was used as a control virus. All viruses were resuspended in Dulbecco's minimal essential medium (DMEM) and injected into the amniotic fluid as described previously [23]. Fetuses were injected at gestation day E15, E16, or E17. Individual fetuses were injected with a final amniotic fluid virus concentration of 10^9 ^pfu/ml. All animals were harvested at 24 hours post-gene transfer.

### Enzyme Assay

Beta galactosidase activity was measured using Applied Biosystems Galacto-Light Plus System (catalog # T1007). Lung tissues were disassociated in the supplied lysis solution, endogenous activity was lowered by heat inactivating for 50 minutes at 48°C and then measured in triplicate. Measurements were made using a tube luminometer (Monolight 2010). Protein concentrations were measured using Bio-Rad Dc Protein Assay (#500–0112).

### Immunofluorescent Microscopy

Lung tissue fixed in 4% paraformaldehyde was embedded in Cryo-Gel and sectioned. Sections were stained with PTHrP (Santa Cruz, sc-20728) and the protein of interest was tagged with Alexa Fluor donkey anti-rabbit 568 (Molecular Probes, A-10042). Nuclei were stained with DAPI and actin was stained with Phalloidin 488 (Molecular Probes, A-12379).

### Statistical methods

Image software SLIDEBOOK was used to capture images and then quantitatively evaluate pixel count. Blinded random fields from taken from 5 independent sections were used. Graph Pad Prism was used for statistic analysis (unpaired, T-test).

## Authors' contributions

JCC, EK, DL, and K–IT are responsible for performing the required laboratory work. JCC, JEL, and K–IT developed the experimental framework and wrote the paper. All authors read and approved the final manuscript.
